# Western Indian Rural Gut Microbial Diversity in Extreme *Prakriti* Endo-Phenotypes Reveals Signature Microbes

**DOI:** 10.3389/fmicb.2018.00118

**Published:** 2018-02-13

**Authors:** Nar S. Chauhan, Rajesh Pandey, Anupam K. Mondal, Shashank Gupta, Manoj K. Verma, Sweta Jain, Vasim Ahmed, Rutuja Patil, Dhiraj Agarwal, Bhushan Girase, Ankita Shrivastava, Fauzul Mobeen, Vikas Sharma, Tulika P. Srivastava, Sanjay K. Juvekar, Bhavana Prasher, Mitali Mukerji, Debasis Dash

**Affiliations:** ^1^Department of Biochemistry, Maharshi Dayanand University, Rohtak, India; ^2^CSIR Ayurgenomics Unit - TRISUTRA (Translational Research and Innovative Science ThRough Ayurgenomics), CSIR-Institute of Genomics and Integrative Biology, New Delhi, India; ^3^G.N. Ramachandran Knowledge Centre for Genome Informatics, CSIR-Institute of Genomics and Integrative Biology, New Delhi, India; ^4^Academy of Scientific and Innovative Research, CSIR-Institute of Genomics & Integrative Biology (IGIB), New Delhi, India; ^5^Vadu Rural Health Program, KEM Hospital Research Centre, Pune, India; ^6^School of Basic Sciences, Indian Institute of Technology, Mandi, India; ^7^Genomics and Molecular Medicine and CSIR-Institute of Genomics and Integrative Biology, New Delhi, India

**Keywords:** Indian gut microbiome, *Prakriti*, precision medicine, ayurgenomics, ayurveda, 16S rRNA gene

## Abstract

Heterogeneity amidst healthy individuals at genomic level is being widely acknowledged. This, in turn, is modulated by differential response to environmental cues and treatment regimens, necessitating the need for stratified/personalized therapy. We intend to understand the molecular determinants of Ayurvedic way (ancient Indian system of medicine) of endo-phenotyping individuals into distinct constitution types termed “*Prakriti,”* which forms the basis of personalized treatment. In this study, we explored and analyzed the healthy human gut microbiome structure within three predominant *Prakriti* groups from a genetically homogenous cohort to discover differentially abundant taxa, using 16S rRNA gene based microbial community profiling. We found Bacteroidetes and Firmicutes as major gut microbial components in varying composition, albeit with similar trend across *Prakriti*. Multiple species of the core microbiome showed differential abundance within *Prakriti* types, with gender specific signature taxons. Our study reveals that despite overall uniform composition of gut microbial community, healthy individuals belonging to different *Prakriti* groups have enrichment of specific bacteria. It highlights the importance of *Prakriti* based endo-phenotypes to explain the variability amongst healthy individuals in gut microbial flora that have important consequences for an individual's health, disease and treatment.

## Introduction

The human microbiome have been shown to have a functional role in the human physiology. Microbial flora and its dynamics are often associated with homeostasis within the body. Systemic characterization of the microbiota for comparison of microbial communities and their contribution to health and disease (Dominguez-Bello and Blaser, 2008; Rosenberg and Zilber-Rosenberg, 2011; Dave et al., 2012) have been carried out by the Human Microbiome Project (HMP) and Metagenomics of the Human Intestinal Tract (MetaHIT) consortium. These studies have provided insights into the composition of microbial community at various anatomical sites (Human Microbiome Project Consortium, 2012; Parfrey and Knight, 2012). The human microbiota has co-evolved closely with its host (Yatsunenko et al., 2012; Moeller et al., 2016) and is modulated by intrinsic and environmental factors. Recent studies have indicated that health and predisposition to various non-infectious diseases of humans are also determined by the genes coded by resident microbiome (Albenberg et al., 2012; Cho and Blaser, 2012; Gordon et al., 2012; Zhang et al., 2015). It is appreciated now that an understanding of human physiology is incomplete without the knowledge of the metagenome. Contemporary approaches have focused on investigating the microbial assemblage of the transient states observed over the course of specific diseases. However, the challenge is to elucidate whether the association between microbial community changes and pathology is causal in nature (Clemente et al., 2012; Haiser and Turnbaugh, 2012). Integrative analysis of human genome, physiology and microbiome will enable better understanding as to whether latter is involved in health and disease. However, there are variables contributed by human host as well as the microbiome, which could confound the observations. For example, the ethnicity and genetic background, age of the individual, dietary and lifestyle habits; all of which have been known to affect the human physiome and shape the microbiome (Fortenberry, 2013; Chong et al., 2015). Studies to identify association between microbial community structure based on ethnicity, diet, gender in healthy individuals, have met with limited success (Chong et al., 2015; Bhute et al., 2016). Absence of definitive patterns has been ascribed to genetic drift as well as population admixture. Therefore, it is a challenge to develop a population based catalog of human microbiome markers for predicting disease predisposition especially for a diverse Indian population. The subcontinent is home to more than one billion people with thousands of endogamous populations from different linguistic lineages and ethnic groups. Along with this diversity, individuals within these population/s have diverse food habits, digestive capabilities, and susceptibility to diseases (Bhute et al., 2016).

Ayurveda, the ancient Indian system of medicine documented and practiced for over 5000 years has an individualized approach to management of health and disease. According to this system, individuals can be classified on the basis of their constitution types termed “*Prakriti*” (Prasher et al., 2008, 2016, 2017; Sethi et al., 2011). *Prakriti* of an individual is determined at the time of birth and remains invariant throughout lifetime. It determines an individual's susceptibility, response to drug, diet and environment as well as prognosis for a disease. *Prakriti* is a consequence of relative proportions of three physiological entities (tridoshas) viz. *Vata* (V), *Pitta* (P), and *Kapha* (K), which govern different functions of transport, metabolism and storage, response to environment and homeostasis in the system. Perturbation of *tridosha* proportions from their homeostatic thresholds leads to disease state. Therefore, individuals based on their dominant proportions of *doshas* are called as *Vata, Pitta, Kapha, Vata-Pitta, Vata-Kapha, Pitta- Kapha, and Vata-Pitta-Kapha Prakriti* types. Amongst the seven constitution types, *Vata, Pitta*, and *Kapha* are the three phenotypic extremes with contrasting disease susceptibilities. Phenotypic assessment of *Prakriti* is carried out on the basis of examination of approximately 150 features comprising of anatomical, physiological, activity related attributes, and psychological parameters. For example, individuals of *Pitta Prakriti* would have better digestion and metabolic capacity whereas *Kapha* would have less and *Vata* with an irregular or unpredictable pattern. Recently, we have also been able to develop predictive models for *Prakriti* that recapitulate the Ayurveda constitution types through phenotypic traits of individuals (Prasher et al., 2008, 2016).

Many of the phenotypic attributes that are being associated with microbiome difference also differ between the constitution types. This includes desire and suitability for different diets, metabolic and digestive patterns, weight gain tendencies, gut motility and excretory patterns (Prasher et al., 2008, 2016). Besides, certain therapeutic modalities unique to Ayurveda that are aimed at maintenance of health and homeostasis lays emphasis on restoration of healthy flora (Prasher et al., 2016, 2017). Earlier, we and other groups have shown that healthy individuals of extreme *Prakriti* types, *Vata, Pitta, and Kapha* comprise 8–10% of a population and exhibit genome wide differences amongst constitution types, albeit from a genetically homogeneous background (Prasher et al., 2008; Aggarwal et al., 2010; Rotti et al., 2014; Govindaraj et al., 2015). These sub-types have underlying differences in genes that modulate pathways for apoptosis, metabolism, hypoxia response, haemostasis, and development. These differences can contribute to inter-individual variability in adaptation to high altitudes, susceptibility to high altitude pulmonary edema or thrombotic outcomes in hypoxia (Prasher et al., 2008; Aggarwal et al., 2010, 2015). Considering the significance of human microbiome in health and diseases, the current study is proposed to analyse gut microbiome in extreme constitution types to explore whether there could be *prakriti* specific microbial assemblage. The study was carried out in a genetically homogenous rural population comprising of healthy individuals of similar age group and dietary habits that were phenotypically stratified on the basis of *Prakriti*.

## Materials and methods

### Volunteer recruitment, *Prakriti* ascertainment, sample collection

The subjects were identified from a rural population in the Pune district of Western India. These participants belong to a cohort from Vadu Health and Demographic Surveillance System (Vadu HDSS) area who have been followed over years by the Vadu Rural Health Program, KEM Hospital Research Centre, Pune. The details of the sampling strategy and recruitment have been described earlier (Tiwari et al., 2017). Predominant *Prakriti* types, which comprises 8–10% of the population, were identified from randomly selected 10,100 individuals between the age group of 18–40 years. After preliminary screening using a questionnaire, 528 self reported healthy individuals were enrolled for detailed *Prakriti* evaluation using a questionnaire and methods developed in our earlier study (Prasher et al., 2008). *Prakriti* screening and clinical assessment was carried out by Ayurveda clinicians and a trained team of field research assistants. Assignment to *Prakriti* groups was carried out by two groups of physicians, one at field site and the other at CSIR-IGIB. Using unsupervised clustering approaches we have recently shown that these *Prakriti* groups form three natural clusters (Tiwari et al., 2017). The enrolled subjects were requested to provide fresh stool samples and it was ensured that they were not under any medication especially antibiotics. Field camps were organized in residential villages in the Vadu HDSS area. Two separate home visits were made by field teams—the first one, 8 days prior to camp to ensure availability of participants and the second, a day prior to camp to provide sterile containers along with the instructions to collect fresh stool samples. Standard operating procedures were strictly adhered to, while collecting samples, their storage at Vadu molecular lab, isolation of DNA, quality assurance and transportation of DNA aliquot to processing lab at CSIR-IGIB. A total of 135 extreme *Prakriti* individuals were identified, namely *Kapha* (*n* = 48), *Pitta* (*n* = 35), and *Vata* (*n* = 52).

The study population is relatively homogeneous in terms of ethnic and linguistic background as well as with respect to dietary and socio-cultural life style. In order to reaffirm the genetic homogeneity of the study population, we have earlier analyzed the genetic relatedness using unlinked and shared panel of 17675 SNP markers with the Indian Genome Variation Consortium (IGVC) diversity panel. Principal Component analysis (PCA) of the genotype data performed using EIGENSOFT 5.0 reaffirmed the homogeneity of the study population (Tiwari et al., 2017). This study has been carried out as per protocols approved by institutional ethics committee at CSIR-Institute of Genomics and Integrative Biology, Delhi and KEM Hospital Research Centre, Pune, India. Fresh stool samples were collected from subjects belonging to predominant *Prakriti* groups (Supplementary Table S1). Metagenomic DNA from stool samples was isolated using QIAamp DNA stool mini kit (Qiagen, Cat. No. 51504, USA).

### 16S rRNA gene amplicon sequencing

We amplified and sequenced V2-V6 region of 16S rRNA gene using metagenomic DNA of 135 individuals, inclusive of 70 females and 65 males using Roche GS FLX+ sequencing technology. At highest stringency of Q40, we got approximately 580 Mb per sequencing run with median read length of 800 bps.

### Raw data processing and community compositional estimates

The Quantitative Insights into Microbial Ecology (QIIME) software package version 1.8.0 (Caporaso et al., 2010) was used to process and analyse raw sequencing data, separately for the male and female datasets. The split_library.py script was used in QIIME as a quality filtering step in which each sample was pre-processed with the maximum allowed one barcode error and two ambiguous bases (Ns). Sequences shorter than 300 or longer than 800 nucleotides with average quality <30 were removed from downstream analysis. In first pass of quality filtering, chimeric sequences were identified and removed by USEARCH 6.1 through QIIME's chimera processing scripts. Reads were clustered into operational taxonomic units (OTUs) with a sequence similarity threshold of 97% using UCLUST v1.2.22q (Edgar, 2010), within QIIME. Reads were assigned to OTUs using a closed reference OTU picking workflow (Caporaso et al., 2010) against GreenGenes 16S rRNA gene database (version 13_8), filtered at 97% sequence identity. In a closed-reference OTU picking, input sequences are aligned to pre-identified taxonomic clusters in a reference database. The input sequence is excluded if it does not match any reference sequence at user defined identity threshold. In further analysis, GreenGenes reference tree and taxonomic assignments were used. Microbial abundance were normalized to generate relative abundance of taxa present in each sample.

### Gut microbiome diversity analysis

Alpha diversity (Shannon diversity and Observed species) for all samples were calculated using QIIME, to estimate species diversity, richness and evenness. Overall taxonomic differences and beta diversity were estimated through Principal Coordinates Analysis (PCoA) based on Bray-Curtis distances, using LabDSV (Roberts, 2013). Results of analyses were visualized using ggplot2 (Wickham, 2016) on R. The taxa of two highly abundant phyla, viz., Firmicutes and Bacteroidetes were removed in QIIME. The alpha diversity (Shannon diversity, richness and evenness) was calculated using “Vegan” package (Dixon, 2003) in R and beta diversity was calculated as mentioned above.

### Estimation of core microbiome and biomarker discovery

Considering the variable nature of metagenomic compositional data, we also performed further analysis only for conserved taxons. Toward this, we estimated core microbial group within the samples with presence in at least 50% of the study samples. Taxonomic classification using alpha and beta diversity were analyzed for the core microbiome as explained above. LEfSe (Segata et al., 2011) was used to identify the microbiological markers associated with *Prakriti* by linear discriminate analysis (LDA) effect size of 2, and for multiclass analysis one-against-all option was used with default parameters (Goecks et al., 2010). Differentially abundant taxons were annotated to their genus and species level through manual Blast (NCBI web server) against the “refseq_rna” database, retaining the highest scoring hit. Since our objective was to identify signature taxa pertaining to *Prakriti* groups, we systematically removed redundancies keeping the OTU with highest LDA score and selected only those differential taxa that were exclusively present in a particular group.

### qPCR validation of *Prakriti* specific microbial enterotypes

qPCR based validation of *Prakriti* specific dominant microbial enterotypes, *Prevotella, Roseburia hominis, Eubacterium rectale, Blautia torques*, and *Blautia obeum* was performed by absolute quantification of 16S rRNA gene copy number using genus specific primers (Supplementary Table S2). Total of 48 samples was used with proportional representation of *Prakriti* types. DNA template concentration for each sample was adjusted to 25 ng/μl. Amplification and detection were performed in a 10 μl reaction [5 μl 2x KAPA SYBR Green PCR Master Mix, 1 μl of each specific primer (10 μM), 1 μl template (25 ng/μl) and 2 μl molecular biology grade water] in triplicate using LC480 Real time PCR system (Roche, Switzerland). Amplification condition include one cycle of activation at 50°C for 2 min and denaturation at 95°C for 3 min, followed by 45 cycles at 95°C for 30 s, 58/64°C for 30 s, 72°C for 40 s; followed by extension at 72°C for 3 min. Melting curve was analyzed for non-specific products at 95°C for 5 s, 65°C for 1 min and 72°C continuous, followed by cooling at 37°C for 3 min. Group specific standard curves was generated from 10-fold serial dilutions of a known concentration of genomic DNA. Average Ct-values of the triplicate was used for estimating 16S rRNA gene copy numbers for each group using standard curves. Percentage abundance of each genus was obtained by calculating ratio of copy number of that genus to that of total bacteria (Eubacteria). Throughout the qPCR, efficiency was maintained above 90% with a correlation coefficient of >0.99. The statistical significance differences for *Prakriti* specific microbial enterotypes were determined by “Wilcoxon Rank Sum” test in R package.

## Results

### Uniform microbial taxonomic distribution and variability in *Prakriti* groups of healthy individuals

Using high throughput sequencing, we obtained 2,699,584 and 2,266,514 reads of 16S rRNA gene from 65 male and 70 female predominant *Prakriti* individuals respectively. Quality filtered raw sequences were taken forward for OTU identification and analyses. QIIME assisted chimera identification using USEARCH resulted in removal of 63,973 and 66,965 sequences from the male and female respectively, with remaining quality filtered set of 2,054,437 and 1,697,708 reads. GreenGenes based OTU identification through closed reference OTU picking protocol of QIIME resulted in identification of 3363 unique OTUs among males and 2882 within females. For stringency, reduced noise in the data and to ensure proper coverage of the entire gut microbial flora, we discarded samples with <4,000 reads. Our final dataset had 1,870,897 and 1,445,312 sequences from 50 male and 63 female subjects. We detected on average, 32,570 and 21,586 OTUs across male and female samples, indicating good coverage of microbial flora, although with variability (σ_male_ = 25621.7 and σ_female_ = 14529.2). To overcome this variability, we normalized the absolute abundance counts to reflect relative abundances.

**Figure 1:**
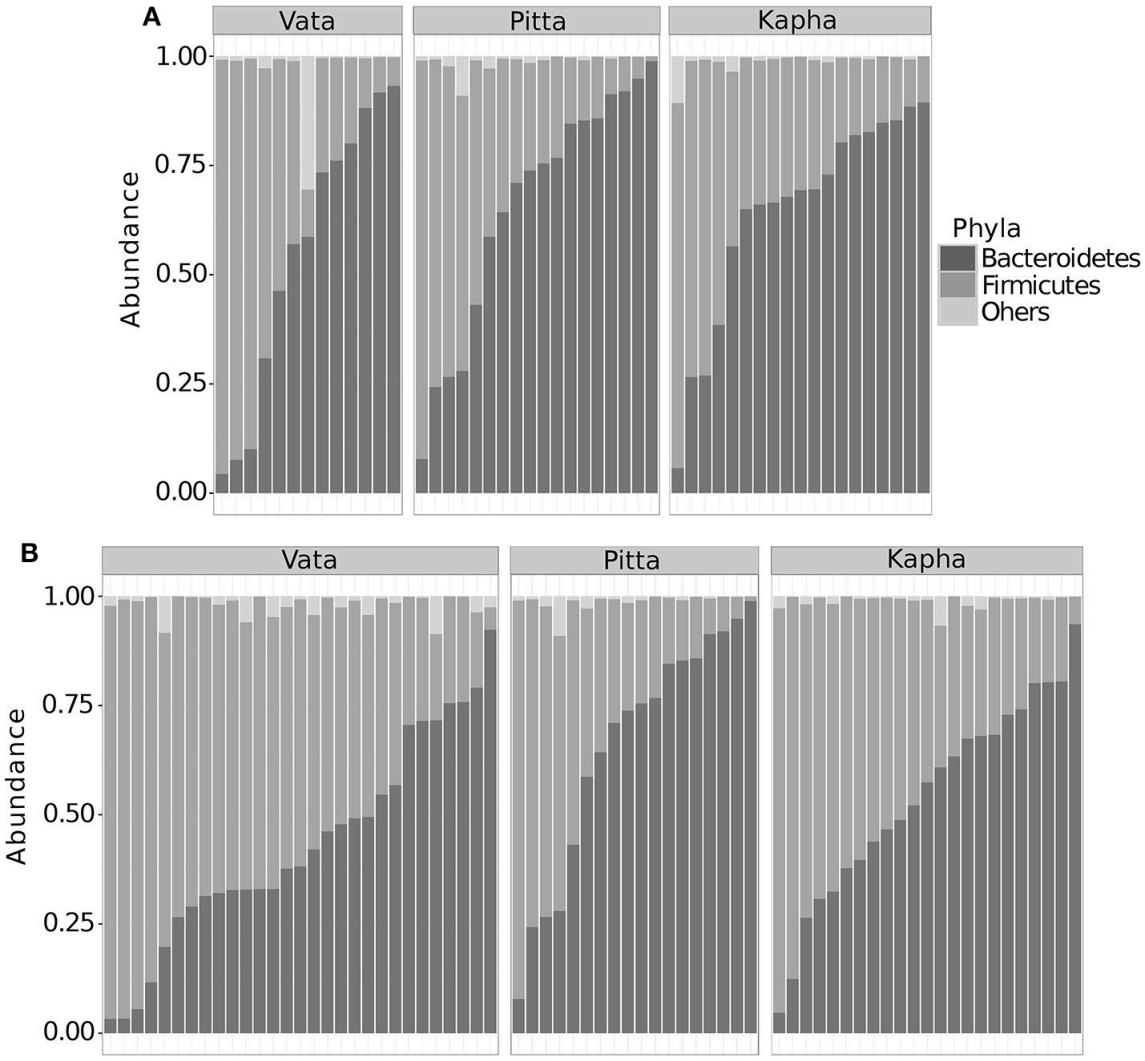
Phyla level taxonomic summary of **(A)** male and **(B)** female gut microbial communities. Subjects have been grouped based on their corresponding *Prakriti*. Figure shows distribution of the two major gut microbial phyla Bacteroidetes and Firmicutes.

**Figure 2:**
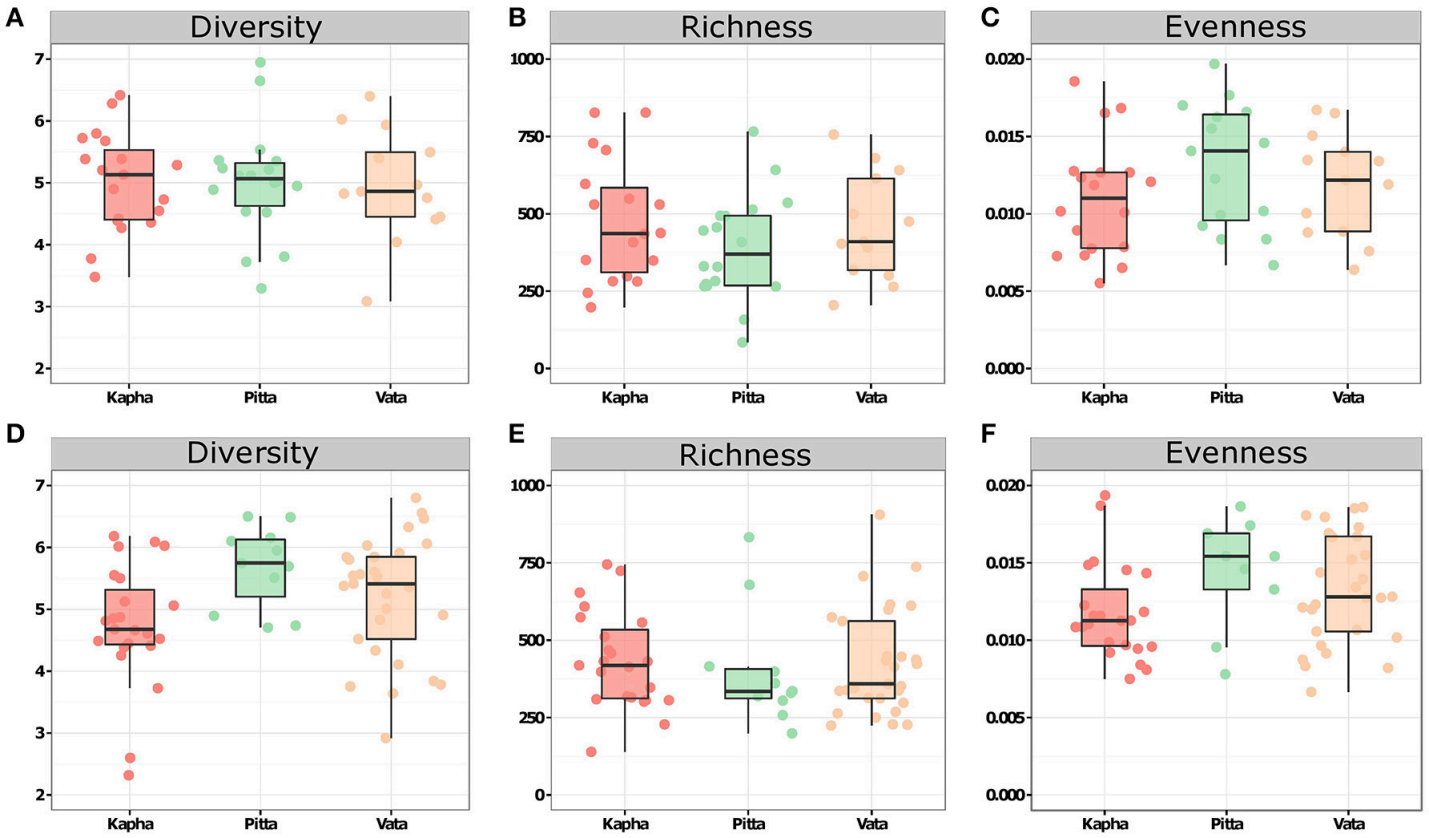
Diversity estimates in gut microbial communities of healthy male and female subjects. **(A)** Diversity (Shannon), **(B)** Richness, and **(C)** Evenness in male samples. **(D)** Diversity (Shannon), **(E)** Richness, and **(F)** Evenness in female samples.

Taxonomic summary shows Bacteroidetes and Firmicutes to be the majority constituents at phylum level for both the male and female groups across *Prakriti* groups, together accounting for more than 98 percent of total abundance (σ_male_ = 4.5%, σ_female_ = 1.9%) (Figure 1). To summarize diversities in individuals and *Prakriti* groups, we used the alpha and beta diversity metrics as introduced by R. H. Whittaker. Alpha diversity captures the richness, evenness and diversity of a sample. Our analyses showed that individuals have non-homogenous composition within their gut microbial assemblage, however with comparable alpha diversity variations across different *Prakriti* (Figure 2). Similarly, our beta diversity analysis to investigate community differences revealed that there was no clustering of individuals with respect to *Prakriti* (Supplementary Figure S1). We made similar observations across both genders. This is in consonance with previous findings of genomic and transcriptomic heterogeneity in healthy individuals (Cho and Blaser, 2012; Schwartz et al., 2012; Zhang et al., 2014). However, it needs to be highlighted that Bacteroidetes and Firmicutes are the two overwhelmingly dominant phyla in gut communities, which may have a masking effect on contributions of specific organisms of different phyla.

In order to access the roles of the lesser abundant taxa in *Prakriti* classifications, we excluded the top two abundant phyla, viz., Bacteroidetes and Firmicutes, and re-estimated the alpha and beta diversity. The alpha diversity within females showed that among the different *Prakriti, Pitta* individuals had lesser diversity and richness but higher evenness than the *Vata* and *Kapha* (Supplementary Figure S2). However, the diversity, richness and evenness was found to be similar in all the three *Prakriti* in the male samples (Supplementary Figure S2). The beta diversity analysis showed no *Prakriti* specific separation of male and female samples in PCoA plot even after the removal of the most highly abundant phyla (Supplementary Figure S2). Despite inter-individual heterogeneity, we observed overlapping variation across the *Prakriti* classes, which most likely is an outcome of similar genetic makeup and lifestyle habits.

### Core microbial community and conserved diversity

**Figure 3:**
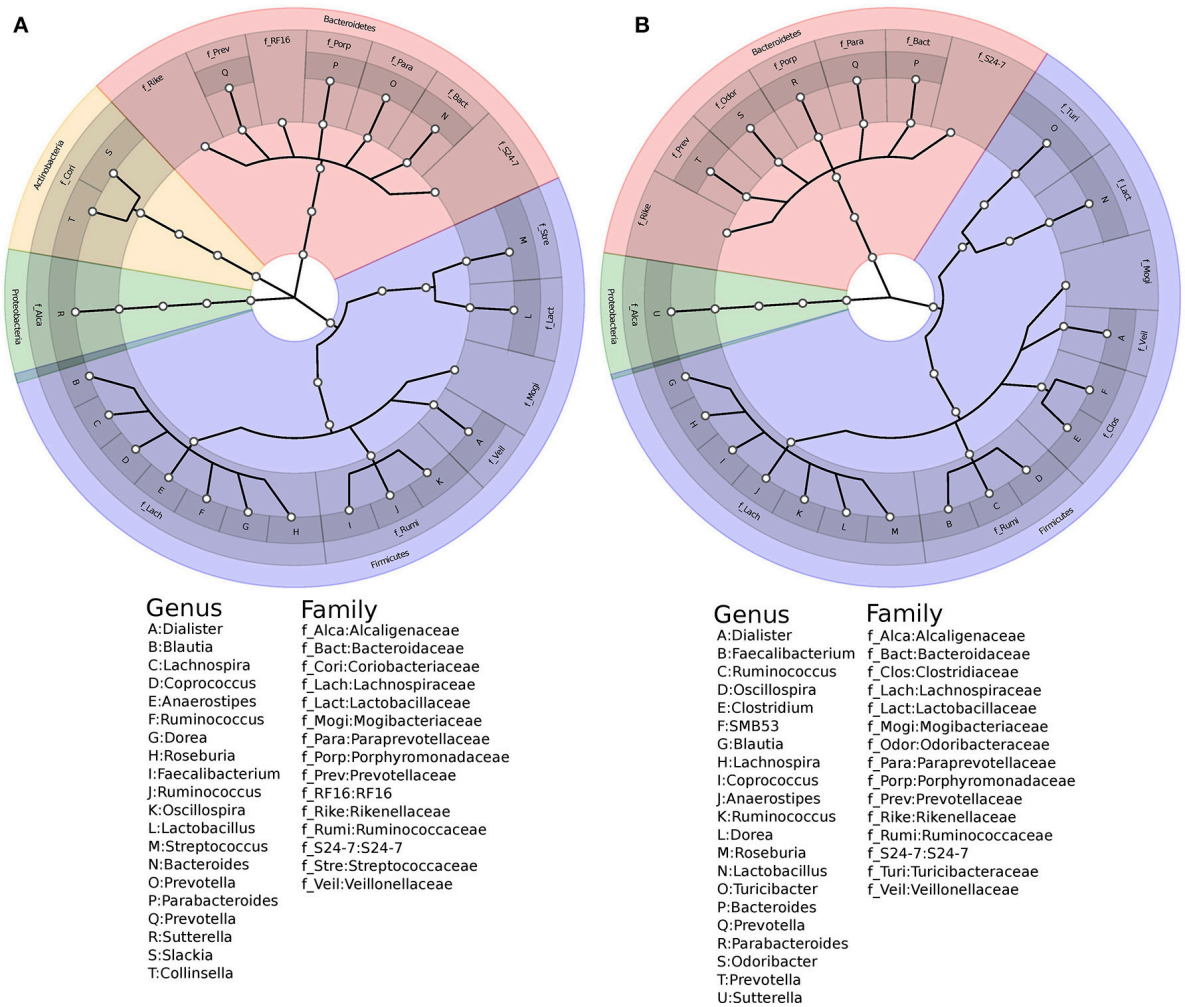
Family and genus that comprise the core microbiome. **(A)** Males and **(B)** Females.

Stable members of a microbial community often modulate physiology of the host-microbial symbiotic system (Tschop et al., 2009; Shade and Handelsman, 2012; D'Ainsworth et al., 2015). The dysbiosis or differential abundance is one of the primary determinants of health and disease spectrum. To investigate this, we estimated core microbiome by qualifying OTUs as core only if their presence was consistent across 50% of all samples. Using in-house custom scripts, we filtered taxons from the male and female groups, to identify 209 and 224 OTUs respectively (Supplementary Table S3). Taxonomic analysis of core groups showed that the phyla Bacteroidetes and Firmicutes follow similar trends of composition as that of the total microbiome. However, the core in female group was comprised of only Bacteroidetes, Firmicutes and Proteobacteria, whereas the core in males had additional members of the family *Coriobacteriaceae* of phylum *Actinobacteria* (Figure 3). We also observed abundant bacterial species to be present across all healthy individuals, thereby occupying majority space within the core microbiome group. Core microbiome also exhibited comparable alpha and beta diversity trends as that of the total microbiome.

### Microbial taxons associated with *Prakriti* types

**Figure 4:**
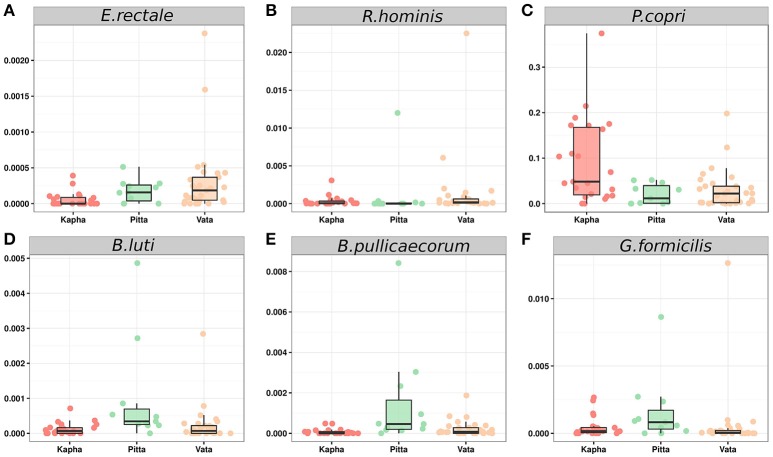
Relative abundances of *Prakriti* specific signature taxons in female subjects. *Eubacteriumrectale* **(A)** and *Roseburia hominis* **(B)** in *Vata*; *Prevotella copri* **(C)** in *Kapha*; *Blautia luti* **(D)**, *Butyricicoccus pullicaecoruma*, **(E)** and *Gemmigerformicilis* **(F)** in *Pitta*.

To control for sparseness associated with gut microbiome profiling, we looked for microbial organisms that were part of the core microbiome while being differentially abundant in a particular *Prakriti* using LEfSe. We performed statistical tests at multiple taxonomic levels and discovered 49 and four taxons across female and male respectively, to be significantly enriched in specific *Prakriti* categories. To control for potential false positives, we manually curated these differentially abundant species to arrive at a final set of 15 and two taxons that were associated with *Prakriti* and had no taxonomic overlap with other *Prakriti* specific bacteria (Table 1). To identify the correct taxonomic details of the signature OTUs, we used sequence alignment methods to identify the most probable candidate at the species level. It is important to note that these specific abundant species are present across most individuals in all *Prakriti* groups, however they are significantly enriched in a particular *Prakriti*, hence termed as signature species (Table 1, Figures 4A–F).

**Table 1:** List of *Prakriti* specific signature taxa with details of their functional importance in the human gut.

**Signature Taxa**	**Gender**	***Prakriti***	**OTUID**	***p*-value**	**LDA**	**Physiological relevance in human gut**	**References**
*Prevotella copri*	Female	*Kapha*	215670	0.006	5.620623	Proinflammatory, onset of rheumatoid arthritis, insulin resistance	Wu et al., 2011; Scher et al., 2013
*Blautia luti*	Female	*Pitta*	178762	0.005	5.19693	Butyrate producers, protect from graft versus host disease, restricts colonization of Vibrio cholera	Hsiao et al., 2014; Eren et al., 2015; Jenq et al., 2015
*Blautia obeum*	Female	*Pitta*	186748	0.018	4.759854	Butyrate producers, protect from graft versus host disease, restricts colonization of Vibrio cholera	Hsiao et al., 2014; Eren et al., 2015; Jenq et al., 2015
*Blautia torques*	Female	*Pitta*	3272764	0.003	4.885697	Butyrate producers, protect from graft versus host disease, restricts colonization of Vibrio cholera	Hsiao et al., 2014; Eren et al., 2015; Jenq et al., 2015
*Butyricicoccus pullicaecorum*	Female	*Pitta*	179826	0.001	5.158494	Butyrate producers, protects from IBS, potential probiotic	Eeckhaut et al., 2013; Geirnaert et al., 2014
*Gemmiger formicilis*	Female	*Pitta*	341024	0.028	4.878518	Induced during CTM treatment of T2D	Xu et al., 2015
*Incertae Sedis Mahella*	Female	*Pitta*	191783	0.026	4.828228	–	–
*Lachnospira eligens*	Female	*Pitta*	176269	0.005	4.822001	–	–
*Bacteroides vulgatus*	Female	*Vata*	184753	0.016	4.753459	Induces insulin resistance, but found to protect from obseity in mice	Ridaura et al., 2013; Pedersen et al., 2016
*Blautia stercoris*	Female	*Vata*	185824	0.018	4.654206	–	–
*Butyrivibrio crossotus*	Female	*Vata*	4349261	0.001	5.137397	Depleted in patients with Chronic Kidney Disease	Barros et al., 2015
*Clostridium indolis*	Female	*Vata*	338992	0.015	5.224559	Carbohydrate metabolism	Biddle et al., 2014
*Eubacterium rectale*	Female	*Vata*	366794	0.001	5.658181	Butyrate producer, depleted during ulcerative colitis	Vermeiren et al., 2012; Machiels et al., 2014; Cockburn et al., 2015; Riviere et al., 2015
*Oscillibacter valericigenes*	Female	*Vata*	175828	0.049	4.617719	*Oscillibacter* related with bacterimia	Sydenham et al., 2014
*Roseburia hominis*	Female	*Vata*	198945	0.011	4.671769	Butyrate producer, depleted during ulcerative colitis	Vermeiren et al., 2012; Machiels et al., 2014; Cockburn et al., 2015; Riviere et al., 2015
*Roseburia inulinivorans*	Male	*Pitta*	199091	0.004	4.526317	Butyrate producer	Scott et al., 2006, 2011
*Fusicatenibacter saccharivorans*	Male	*Vata*	183401	0.045	4.705687	–	–

The *Pitta* females have over representation of seven species, out of which three belong to the genus *Blautia*—*Blautia luti, B. obeum*, and *B. torques* (Figure 4D). *Blautia* is a newly classified genera in the order *Clostridiales*, and its species constitute a major fraction of the gut flora, often responsible for conversion of carbon and hydrogen to acetate (Liu et al., 2008). *Blautia*, a commensal group of bacteria associated with nutrition processing for the host (Eren et al., 2015) is also associated with protection from graft versus host disease (Jenq et al., 2015) and restricts colonization of *Vibrio cholera*, thereby aiding in recovery from disease (Hsiao et al., 2014). Few *Blautia* species have increased levels during diseases like irritable bowel syndrome (IBS), though their role in the pathology is yet to be ascertained (Rajilic-Stojanovic and de Vos, 2014; Taverniti and Guglielmetti, 2014). *Pitta* females also showed overabundance of *Butyricicoccus pullicaecoruma*, a butyrate producing beneficial bacteria (Figure 4E), which has a protective effect against IBS (Eeckhaut et al., 2013) and is being considered as a potential probiotic (Geirnaert et al., 2014). Another bacterium specifically enriched in *Pitta* females was *Gemmiger formicilis* (Figure 4F), a beneficial bacteria which has been shown to be induced by a Chinese traditional medicine treatment for type 2 diabetes (Xu et al., 2015). Additionally, *Incertae Sedis Mahella* and *Lachnospira eligens* were significantly overabundant in female subjects of *Pitta Prakriti*, however their role in the human gut remain unclear (Supplementary Figure S3).

*Kapha* females were characterized by overabundance of *Prevotella copri* (Figure 4C), a species of bacteria commonly associated with a plant rich diet (Wu et al., 2011). It is also shown to be strongly correlated with inflammation and rheumatoid arthritis (RA) (Scher et al., 2013). Very recently, *P. copri* was found to induce insulin resistance in humans, resulting in increased glucose insensitivity through biosynthesis of branched-chain amino acids (BCAA) (Pedersen et al., 2016).

In *Vata* females, we observed significant abundance of *Bacteroides vulgatus*, which along with *Prevotella copri*, is shown to induce insulin resistance (Pedersen et al., 2016). Interestingly, studies in mice have also highlighted its protective effect in obesity and related metabolic disorders (Ridaura et al., 2013). Signature species of *Vata* females included *E. rectale* and *R. hominis* (Figures 4A,B), butyrate producers. They are believed to be beneficial for the gut health as they have been observed to be depleted during ulcerative colitis in independent studies, similar to *Faecalibacterium prausnitzii* (Vermeiren et al., 2012; Machiels et al., 2014; Cockburn et al., 2015; Riviere et al., 2015). *Vata* females also show enrichment of *Butyrivibrio crossotus* which was found to be depleted in chronic kidney disease patients (Barros et al., 2015). Relative overabundance of these species suggest a healthy intestinal flora in the *Vata* subjects, however we also note excess of *Oscillibacter valericigenes* which belongs to the genera *Oscillibacter* that have been reported in a case of bacterimia (Sydenham et al., 2014). We identified two other signature species, *Blautia stercoris* and *Clostridium indolis* (Supplementary Figure S3), whose role in the human gut is not clear, though *Clostridium indolis* is believed to be involved in carbohydrate metabolism (Biddle et al., 2014). This indicates the importance of this species in gut and its abundance dynamics can have a protective as well as an adverse effect.

Though we detected more OTUs among male subjects (Supplementary Figure S4), we found very few *Prakriti* specific signature microbes that qualified our stringent analysis threshold. We observed two signature bacterial species, *Roseburia inulinivorans* and *Fusicatenibacter saccharivorans* belonging to *Pitta* and *Vata* subjects respectively. Though *R. inulinivorans* has been characterized to be a butyrate bacteria of potentially beneficial contribution to the intestinal health (Scott et al., 2006), there is limited information with respect to mechanism underlying as to how overabundance of these two species might affect the human gut. Our analysis suggests that the male gut communities are relatively more homogenous as compared to female counterpart, which may have resulted in detection of fewer differentially abundant species.

### qPCR validation of *Prakriti* specific microbial enterotypes

**Figure 5:**
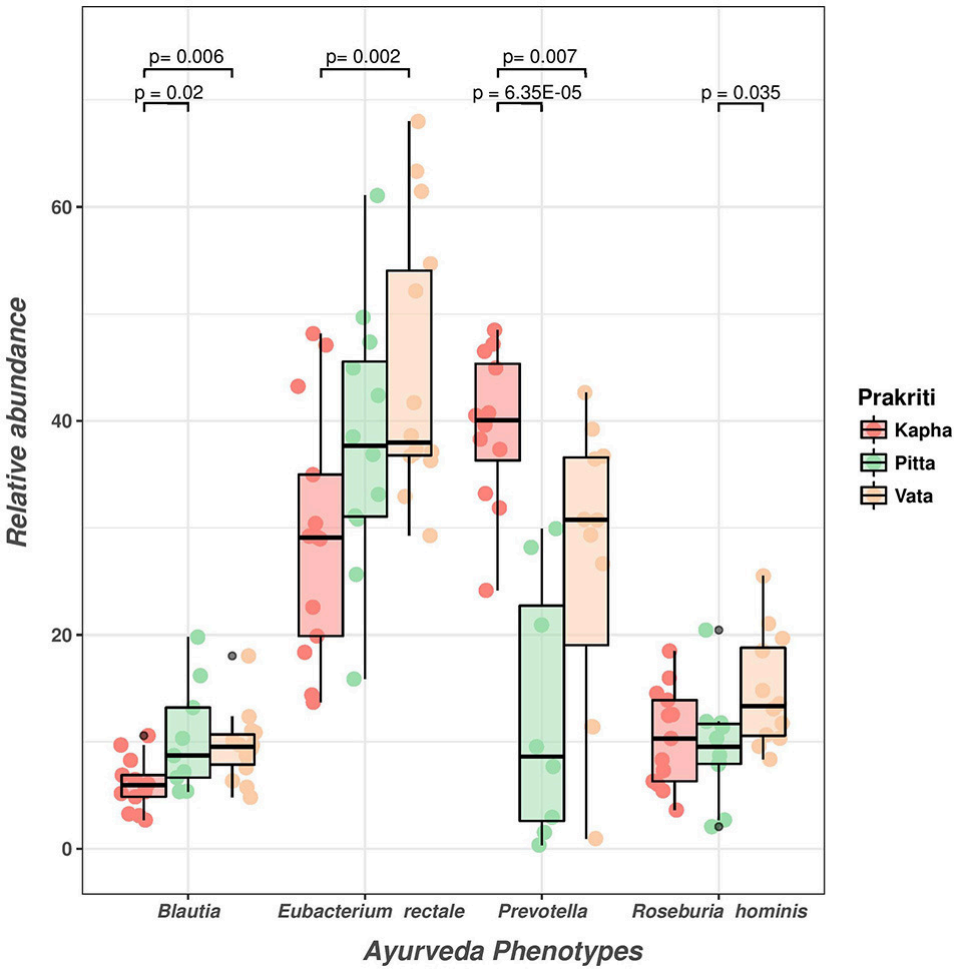
qPCR analysis to validate relative abundance of differentially abundant microbial enterotypes in *Vata, Pitta*, and *Kapha*. Statistical significance of *Prakriti* specific microbial enterotypes were determined by “Wilcoxon Rank Sum” test.

To validate the 16S rRNA amplicon based identification of *Prakriti* specific microbial enterotypes, we carried out qPCR assays for absolute quantification of 16S rRNA gene copy number of differentially abundant microbial enterotypes in the study subjects (Supplementary Table S4). Relative abundance analysis of microbial enterotypes in different *Prakriti* has re-affirmed the insights from the 16S rRNA gene sequencing analysis (Figure 1). The differential abundance of signature species within each *Prakriti* type also show statistically significant difference based on qPCR. *Prevotella* group was significantly abundant in *Kapha* females compared to *Pitta* (p-0.0000635) and *Vata* (p-0.007), while *Eubacterium rectale* & *Roseburia* group was found significantly enriched (p-0.002 and p-0.035) within *Vata* females. Simultaneously, *Blautia* sp. was statistically enriched (p-0.006, p-0.02) within *Pitta* females (Figure 5). It would be important to understand and explore the functional role of these microbes vis-à-vis *Prakriti* types in subsequent studies.

## Discussion

Human health is the manifestation of a perpetual interaction between the human body and its surroundings. Increasing evidence suggests an underlying molecular heterogeneity in healthy individuals, which result in differential response to disease and their treatments. Over the past decade, we have increasingly realized the importance of microbiome and its role in human health (Cho and Blaser, 2012; Zhang et al., 2014). Advances in genomic technologies have facilitated significant discoveries in the area of metagenomics and some have already made their way into clinical practices. Thus it is important to understand the variability in the microbial flora among healthy individuals and its role in disease predisposition, protection and prognostic markers (Parfrey and Knight, 2012). Ayurveda begins with identification of an individual's intrinsic constitution “*Prakriti,”* whereas diseased state “*Vikriti”* is considered to be a deviation from the baseline. The restorative regimen addresses the cause and baseline in an individualized manner and hence closely resembles precision medicine (Prasher et al., 2008, 2017; Dey and Pahwa, 2014).

In this study, we examined the gut microbial community of 113 male and female volunteers with predominant *Prakriti* phenotypes from the Vadu HDSS population (western part of India), in an effort to catalog the gut microbial diversity among healthy individuals. Our results show that genetically homogenous population with similar cultural and dietary habits, can have subtle yet important variations within the gut microbial community. Alpha and beta diversity analyses revealed marked differences in community composition among subjects, albeit, at *Prakriti* level, we observe a homogenous gut microbiome across male and female. Less than one-tenth of the total flora show conservation in each group, indicating a dynamic microbiome in a homogenous population. We queried for *Prakriti* associated differences in the core microbiome to avoid artificial abundance differences that may arise due to sampling bias. The core microbiome resembled that of the total set, with Bacteroidetes and Firmicutes accounting for majority of the phyla detected. We investigated the core microbiome for both genders to search for *Prakriti* associated taxons representative of true differential abundance. Our analysis showed two OTUs to be differentially abundant across *Prakriti* categories in males, whereas females had 15 taxonomic groups, out of 209 and 224 OTUs respectively, which formed the core microbiome.

Our investigation across both genders found microbial taxa indicative of a healthy gut flora reiterating the health status of our study volunteers. The *Pitta* individuals showed enrichment of several butyrate producing microbes, which have been shown to be protective against inflammation, IBS (Geirnaert et al., 2014) and graft-versus-host-disease (Jenq et al., 2015). These suggest that *Pitta* have a robust flora and a healthier gut. Amongst the three extremes, *Pitta Prakriti* has been described to have good digestion and metabolism capacity, regular bowel habit with tendencies for loose motions (Prasher et al., 2008, 2016; Dey and Pahwa, 2014). Additionally, *Pitta* individual are more prone to inflammation (Juyal et al., 2012). Since the study has been carried out on healthy individuals, the presence of bacteria that are protective against inflammation and disease like IBS might suggest their role in maintaining homeostasis. These observations also corroborate with our earlier reported observations of higher expression of immune response genes in *Pitta* compared to *Vata* (Scott et al., 2006).

On the contrary, we observed significant overabundance of *P. copri* among female *Kapha* individuals. *Prevotella* and *P. copri* specifically have been found to compromise host health, and have been associated with rheumatoid arthritis (Juyal et al., 2012; Scher et al., 2013) and insulin resistance (Pedersen et al., 2016). Phenotypes associated with insulin resistance like obesity, susceptibility for type 2 diabetes and atherosclerosis have been described for *Kapha Prakriti* (Prasher et al., 2008; Govindaraj et al., 2015; Doddoli et al., 2016). Besides, our earlier study has also revealed higher levels of lipids in *Kapha* individuals compared to other *Prakriti* types (Prasher et al., 2008; Doddoli et al., 2016). The enrichment of *Prevotella* in *Kapha* might to some extent explain the descriptions of *Prakriti*. This provides an opportunity toward in-depth investigation of gut flora of *Kapha* individuals to assess potential predisposition to these disease states. *Vata* individuals showed a mix of beneficial bacteria and otherwise in their gut flora. In addition to presence of hostile organisms like *B. vulgatus* and *Oscillibacter valericigenes*, we also detected several anti-inflammatory butyrate producing species like *Eubacterium rectale* and *R. hominis*. These observations indicate toward *Vata* individuals being predisposed to health risks pertaining to presence of detrimental microbes, however it also has enrichment of several beneficial bacteria. The combination of non-beneficial and protective species in *Vata* might be able to fine balance health, as is visible in healthy subjects. However, change in their proportions might lead to different outcomes to which *Vata* individuals may be susceptible. *Vata* individuals have been described to have irregular and unpredictable digestion, metabolism and bowel functions (Prasher et al., 2008, 2017; Dey and Pahwa, 2014). They have also been shown to have lower immune responses in our earlier study (Prasher et al., 2008; Dey and Pahwa, 2014). Preventive regimes may be targeted toward maintenance and enhancement of healthy flora specifically in these groups of individuals, highlighting the importance of personalized approach in preventive medicine based on this analysis.

## Conclusions

Using genomic techniques and the principles of Ayurveda, our group has previously elucidated the link between adaptation to low oxygen environment, high altitude pulmonary edema and *Prakriti*. In this study, we embarked to measure the variability in the gut metagenome of healthy individuals and its potential effect on disease vulnerability and natural protection. We discovered several important bacterial species with beneficial as well as detrimental association with human health, being selectively enriched in the gut flora of healthy individuals. Insights obtained from this study provide fundamental understanding of underlying metagenomic heterogeneity and its potential application toward personalized therapy.

## Data availability

NGS sequence reads for samples included in this study can be accessed using the following link https://figshare.com/s/e981faa54cc3347999d9.

## Ethics statement

This study was carried out in accordance with the recommendations of Indian Council of Medical Research, India guidelines for biomedical research, with written informed consent from all subjects. All subjects gave written informed consent in accordance with the Declaration of Helsinki. The protocol was approved by the Institutional Human ethics committees of K.E.M. Hospital and Research Center, Pune as well as Institute of Genomics and Integrative Biology, Delhi, India.

## Author contributions

DD, MM, and BP designed the project. RuP, DA, BG, AS, and SaJ performed volunteer recruitment and sample collection. NC, VA, and RaP performed experiments and NGS sequencing. AM, SG, NC, RaP, TS, FM, and VS performed data analyses. SwJ, NC, MV, performed qPCR experiments and analysis. AM, SG, NC, MV, and RaP wrote the manuscript; MM, DD, and BP reviewed the data and manuscript. All authors have read and approved the manuscript.

### Conflict of interest statement

The authors declare that the research was conducted in the absence of any commercial or financial relationships that could be construed as a potential conflict of interest.

## Supplementary material

Supplementary Figure S1 PCoA plot of beta diversity calculated using Bray-Curtis distance for **(A)** males and **(B)** female samples.

Supplementary Figure S2 Diversity panel of *Prakriti* after removing two highly abundant phyla, viz., Firmicutes and Bacteroidetes. **(A)** Diversity (Shannon), **(B)** Richness, and **(C)** Evenness in male samples. **(E)** Diversity (Shannon), **(F)** Richness, and **(G)** Evenness in female samples. PCoA plot of beta diversity calculated using Bray-Curtis distance **(D)** in males and **(H)** in female samples.

Supplementary Figure S3 Relative abundances of *Prakriti* specific signature taxons *Bacteroides vulgatus* **(A)**; *Blautia obeum* **(B)**; *Blautia stercoris* **(C)**; *Blautia torques* **(D)**; *Butyrivibrio crossotus* **(E)**; *Clostridium indolis* **(F)**; *Incertae Sedis Mahella* **(G)**; *Lachnospira eligens* **(H)**; *Oscillibacter valericigenes* **(I)** in female subjects.

Supplementary Figure S4 Relative abundances of *Prakriti* specific signature taxons i.e., *Roseburia inulinivorans* **(A)** and *Fusicatenibacter saccharivorans* **(B)** in male subjects.

Supplementary Table S1 Details of volunteers enrolled in this study.

Supplementary Table S2 List of Primers used for qPCR analysis.

Supplementary Table S3 Core Microbiome in male and female groups.

Supplementary Table S4 qPCR analysis data to validate relative abundance of differentially abundant microbial enterotypes in *Vata, Pitta*, and *Kapha*.
