# Supplementary figures and images for: Western Indian Rural Gut Microbial Diversity in Extreme Prakriti Endo-Phenotypes Reveals Signature Microbes
Source: Front Microbiol. 2018 Feb 13;9:118. doi: 10.3389/fmicb.2018.00118 (PMC5816807; doi:10.3389/fmicb.2018.00118)

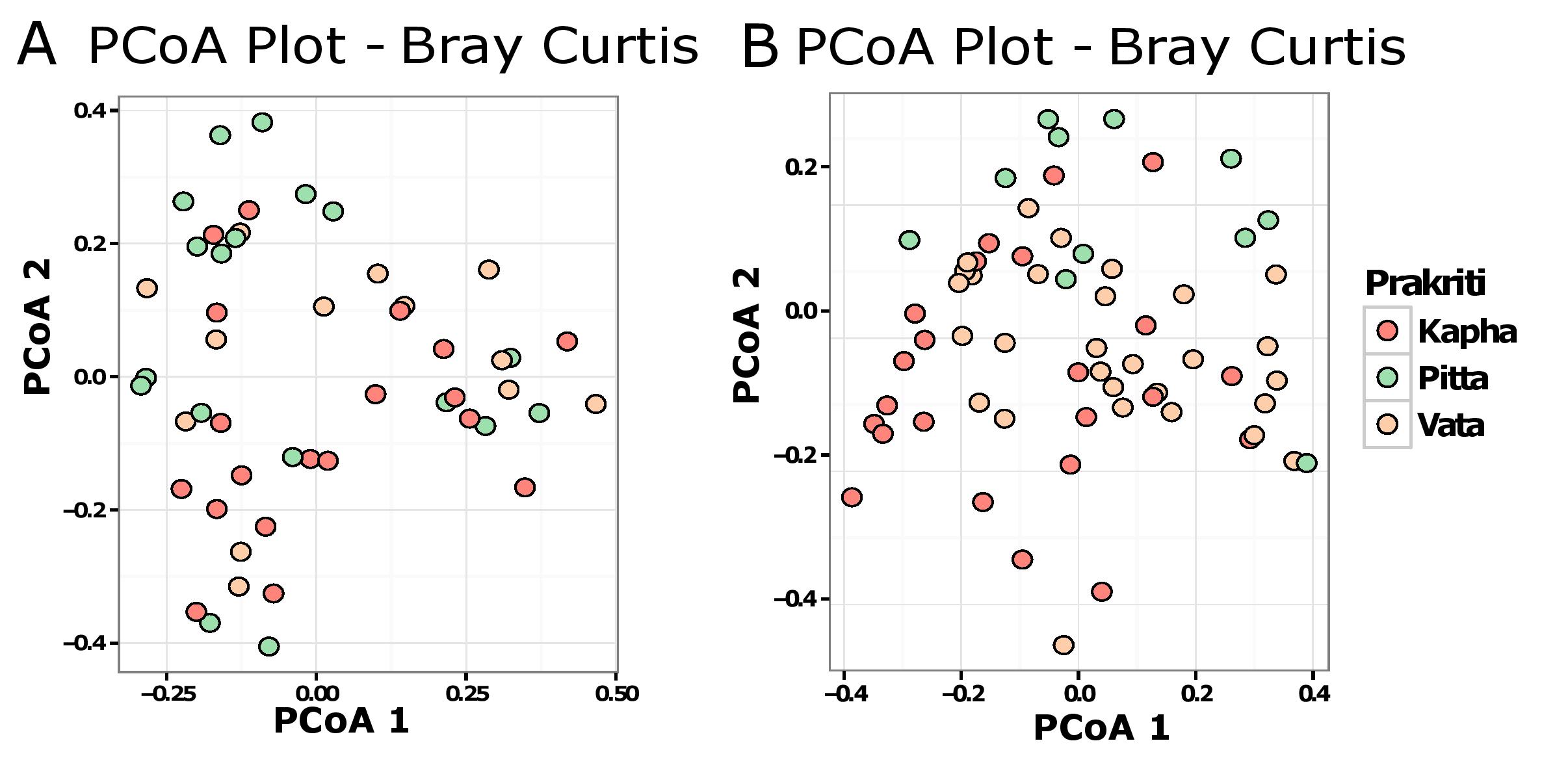

Supplement: Supplementary Figure S1 — PCoA plot of beta diversity calculated using Bray-Curtis distance for (A) males and (B) female samples. [file Image1.JPEG]

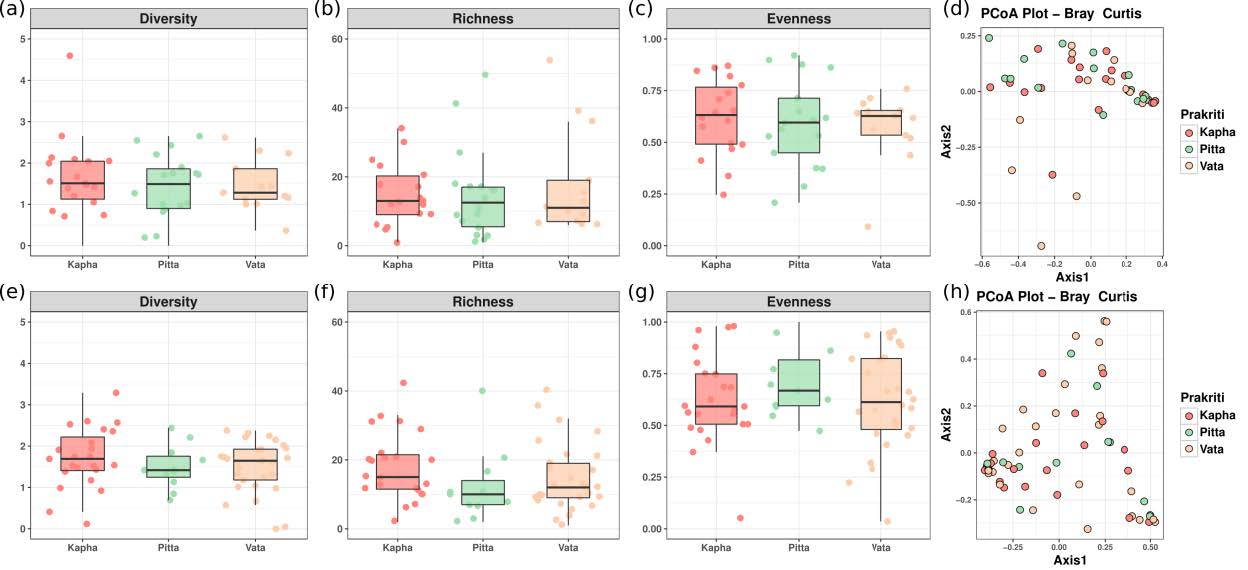

Supplement: Supplementary Figure S2 — Diversity panel of Prakriti after removing two highly abundant phyla, viz., Firmicutes and Bacteroidetes. (A) Diversity (Shannon), (B) Richness, and (C) Evenness in male samples. (E) Diversity (Shannon), (F) Richness, and (G) Evenness in female samples. PCoA plot of beta diversity calculated using Bray-Curtis distance (D) in males and (H) in female samples. [file Image2.JPEG]

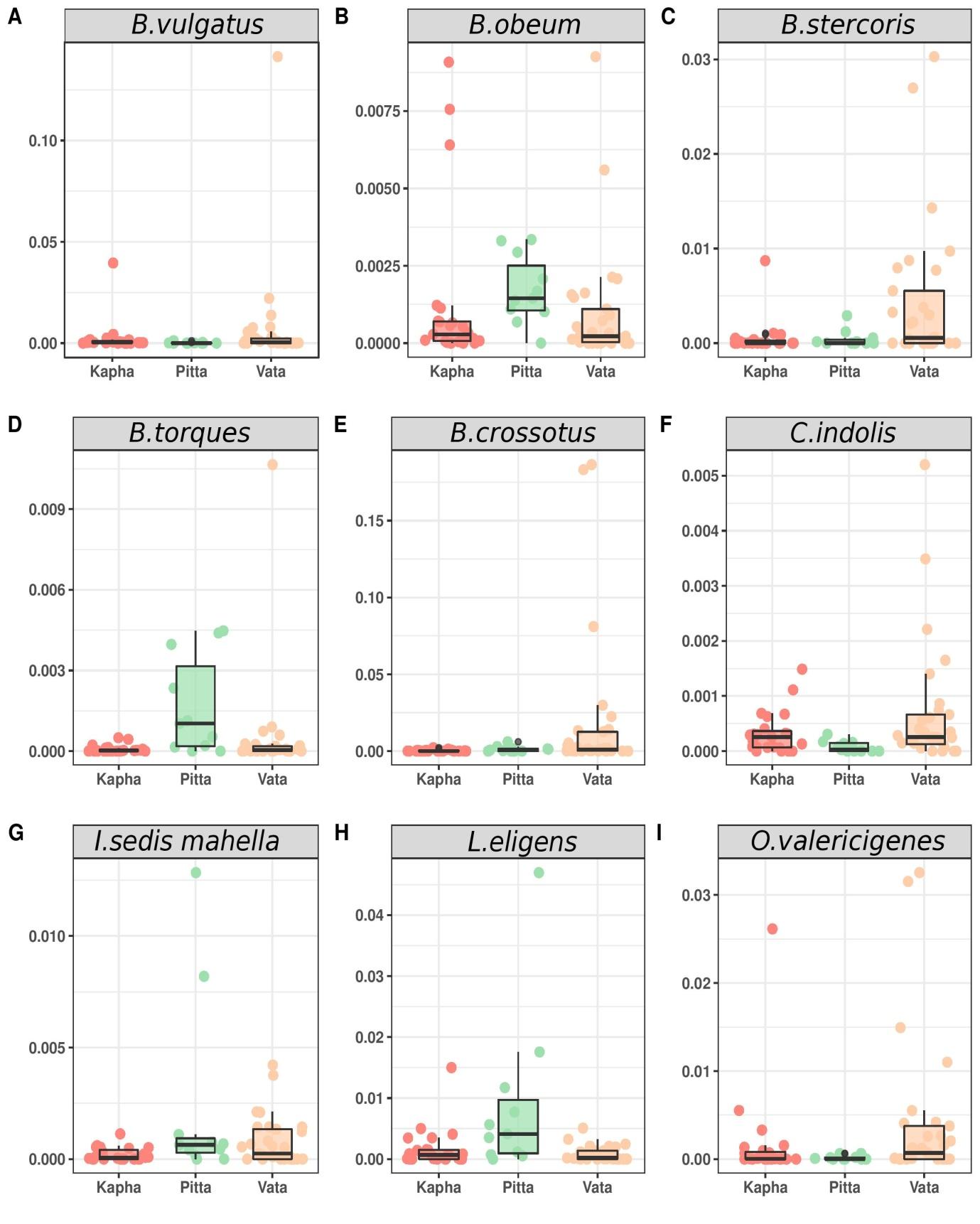

Supplement: Supplementary Figure S3 — Relative abundances of Prakriti specific signature taxons Bacteroides vulgatus (A); Blautia obeum (B); Blautia stercoris (C); Blautia torques (D); Butyrivibrio crossotus (E); Clostridium indolis (F); Incertae Sedis Mahella (G); Lachnospira eligens (H); Oscillibacter valericigenes (I) in female subjects. [file Image3.JPEG]

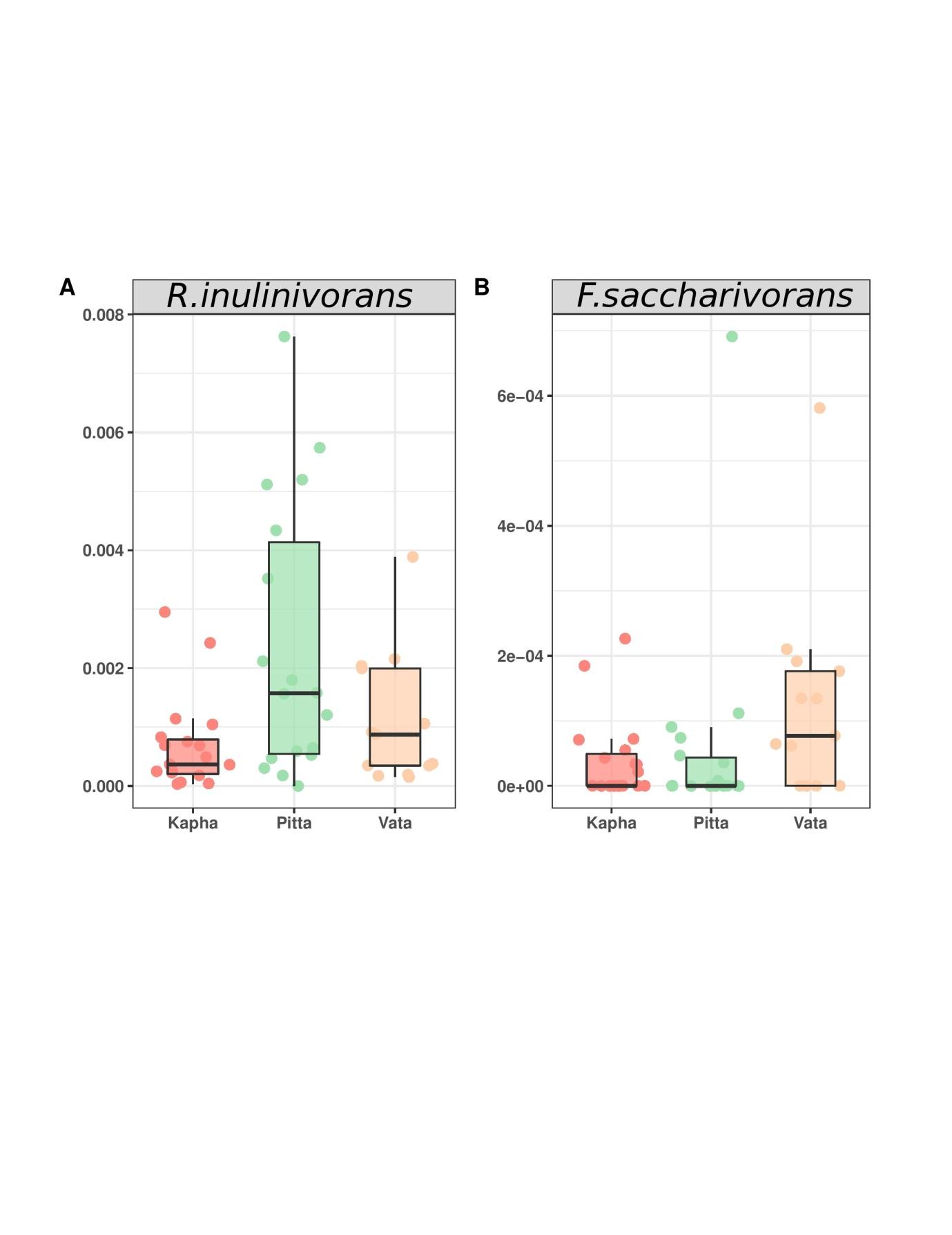

Supplement: Supplementary Figure S4 — Relative abundances of Prakriti specific signature taxons i.e., Roseburia inulinivorans (A) and Fusicatenibacter saccharivorans (B) in male subjects. [file Image4.JPEG]
