# Supplementary material for: Western Indian Rural Gut Microbial Diversity in Extreme Prakriti Endo-Phenotypes Reveals Signature Microbes
Source: Front Microbiol. 2018 Feb 13;9:118. doi: 10.3389/fmicb.2018.00118 (PMC5816807; doi:10.3389/fmicb.2018.00118)
Supplement: Supplementary Table S1 — Details of volunteers enrolled in this study. [file Table1.DOCX]

**SUPPLEMENTARY TABLES**

**ST1 Table. Details of 135 volunteers enrolled in this study.**

**_______________________________**

**Sample ID Prakriti Gender**

**_______________________________**

| **ID** | **Gender** | **Prakriti** |
| --- | --- | --- |
| TR-WIEE-3100 | Female | Kapha |
| TR-DDEB-6681 | Female | Kapha |
| TR-FEEP-4964 | Female | Kapha |
| TR-VVEP-0074 | Female | Kapha |
| TR-EDEF-9625 | Female | Kapha |
| TR-FDEF-4635 | Female | Kapha |
| TR-WWEF-3345 | Female | Kapha |
| TR-EVEF-9075 | Female | Kapha |
| TR-FIEF-4175 | Female | Kapha |
| TR-FEEW-4906 | Female | Kapha |
| TR-UEEW-7906 | Female | Kapha |
| TR-FVEW-4016 | Female | Kapha |
| TR-BUEW-8746 | Female | Kapha |
| TR-HIEW-2156 | Female | Kapha |
| TR-PFEW-5486 | Female | Kapha |
| TR-UBEW-7886 | Female | Kapha |
| TR-VWEH-0327 | Female | Kapha |
| TR-IEEH-1957 | Female | Kapha |
| TR-UEEH-7957 | Female | Kapha |
| TR-EEEH-9997 | Female | Kapha |
| TR-DIEI-6138 | Female | Kapha |
| TR-WPEI-3598 | Female | Kapha |
| TR-EPEI-9598 | Female | Kapha |
| TR-VEEV-0919 | Female | Kapha |
| TR-VFEP-0464 | Female | Pitta |
| TR-DPEP-6574 | Female | Pitta |
| TR-DUEP-6774 | Female | Pitta |
| TR-DVEF-6005 | Female | Pitta |
| TR-DPEF-6535 | Female | Pitta |
| TR-IWEW-1356 | Female | Pitta |
| TR-UDEW-7686 | Female | Pitta |
| TR-FDEH-4607 | Female | Pitta |
| TR-WPEH-3527 | Female | Pitta |
| TR-UWEH-7347 | Female | Pitta |
| TR-FBEH-4877 | Female | Pitta |
| TR-FEEI-4958 | Female | Pitta |
| TR-IUEV-1719 | Female | Pitta |
| TR-HUEV-2789 | Female | Pitta |
| TR-EPED-9583 | Female | Vata |
| TR-FUEP-4724 | Female | Vata |
| TR-PUEP-5784 | Female | Vata |
| TR-UWEF-7305 | Female | Vata |
| TR-UFEF-7415 | Female | Vata |
| TR-UPEF-7535 | Female | Vata |
| TR-HBEF-2865 | Female | Vata |
| TR-BFEW-8406 | Female | Vata |
| TR-VEEW-0936 | Female | Vata |
| TR-FDEW-4646 | Female | Vata |
| TR-IUEW-1746 | Female | Vata |
| TR-BHEW-8276 | Female | Vata |
| TR-DBEW-6876 | Female | Vata |
| TR-EIEW-9186 | Female | Vata |
| TR-FWEH-4307 | Female | Vata |
| TR-BFEH-8417 | Female | Vata |
| TR-HEEH-2947 | Female | Vata |
| TR-VVEH-0057 | Female | Vata |
| TR-BIEH-8157 | Female | Vata |
| TR-IVEH-1087 | Female | Vata |
| TR-FUEH-4787 | Female | Vata |
| TR-UFEH-7497 | Female | Vata |
| TR-UUEI-7758 | Female | Vata |
| TR-BUEI-8758 | Female | Vata |
| TR-EEEI-9958 | Female | Vata |
| TR-IIEI-1168 | Female | Vata |
| TR-FWEI-4398 | Female | Vata |
| TR-UDEV-7629 | Female | Vata |
| TR-WIEV-3139 | Female | Vata |
| TR-DUEV-6759 | Female | Vata |
| TR-VVEV-0079 | Female | Vata |
| TR-BBEV-8879 | Female | Vata |
| TR-DWEE-6300 | Male | Kapha |
| TR-PHEE-5250 | Male | Kapha |
| TR-VDEE-0680 | Male | Kapha |
| TR-DDEE-6680 | Male | Kapha |
| TR-UHEE-7290 | Male | Kapha |
| TR-UHEB-7211 | Male | Kapha |
| TR-HEEB-2921 | Male | Kapha |
| TR-IDEB-1651 | Male | Kapha |
| TR-BDEB-8681 | Male | Kapha |
| TR-BWEB-8391 | Male | Kapha |
| TR-DPEU-6512 | Male | Kapha |
| TR-DEEU-6942 | Male | Kapha |
| TR-IWEU-1382 | Male | Kapha |
| TR-DWEU-6382 | Male | Kapha |
| TR-IDEU-1692 | Male | Kapha |
| TR-HVED-2023 | Male | Kapha |
| TR-VHED-0223 | Male | Kapha |
| TR-BPED-8523 | Male | Kapha |
| TR-UIED-7133 | Male | Kapha |
| TR-HFEP-2434 | Male | Kapha |
| TR-FDEP-4664 | Male | Kapha |
| TR-BBEP-8894 | Male | Kapha |
| TR-UUEF-7735 | Male | Kapha |
| TR-EFEH-9467 | Male | Kapha |
| TR-WFEE-3420 | Male | Pitta |
| TR-BWEE-8360 | Male | Pitta |
| TR-IPEE-1590 | Male | Pitta |
| TR-EEEB-9901 | Male | Pitta |
| TR-IPEB-1521 | Male | Pitta |
| TR-VVEB-0061 | Male | Pitta |
| TR-EBEB-9861 | Male | Pitta |
| TR-VHEB-0291 | Male | Pitta |
| TR-UDEU-7602 | Male | Pitta |
| TR-BEEU-8912 | Male | Pitta |
| TR-UUEU-7752 | Male | Pitta |
| TR-UVEU-7082 | Male | Pitta |
| TR-HEEU-2982 | Male | Pitta |
| TR-BDED-8643 | Male | Pitta |
| TR-DVEP-6004 | Male | Pitta |
| TR-FIEP-4114 | Male | Pitta |
| TR-VHEP-0214 | Male | Pitta |
| TR-WDEP-3614 | Male | Pitta |
| TR-HFEP-2484 | Male | Pitta |
| TR-HWEF-2325 | Male | Pitta |
| TR-IHEW-1226 | Male | Pitta |
| TR-FWEE-4300 | Male | vata |
| TR-HPEE-2500 | Male | Vata |
| TR-HUEB-2761 | Male | Vata |
| TR-EDEB-9671 | Male | vata |
| TR-IPEU-1512 | Male | Vata |
| TR-IUEU-1742 | Male | Vata |
| TR-PPED-5583 | Male | Vata |
| TR-VUEP-0724 | Male | Vata |
| TR-FIEP-4134 | Male | Vata |
| TR-VHEP-0234 | Male | Vata |
| TR-HUEP-2754 | Male | Vata |
| TR-UUEP-7784 | Male | Vata |
| TR-PBEP-5884 | Male | vata |
| TR-UFEF-7405 | Male | Vata |
| TR-IDEF-1615 | Male | Vata |
| TR-BDEF-8665 | Male | Vata |
| TR-PPEF-5585 | Male | Vata |
| TR-EHEW-9286 | Male | Vata |
| TR-BPEI-8528 | Male | Vata |
| TR-EEEV-9909 | Male | vata |
