# Supplementary material for: Western Indian Rural Gut Microbial Diversity in Extreme Prakriti Endo-Phenotypes Reveals Signature Microbes
Source: Front Microbiol. 2018 Feb 13;9:118. doi: 10.3389/fmicb.2018.00118 (PMC5816807; doi:10.3389/fmicb.2018.00118)
Supplement: Supplementary Table S2 — List of Primers used for qPCR analysis. [file Table2.DOCX]

| **Primer ID** | **Primer Sequence**  **5`P-----------------------------3`OH** | **Microbial Targets** |
| --- | --- | --- |
| **Prev_F** | CACCAAGGCGACGATCA | *Prevotella* |
| **Prev_R** | GGATAACGCCYGGACCT | *Prevotella* |
| **Ros_F** | TACTGCATTGGAAACTGTCG | Roseburia *sp.* |
| **Ros_R** | CGGCACCGAAGAGCAAT | Roseburia *sp.* |
| **ClEub F** | CGGTACCTGACTAAGAAGC | *Eubacterium rectale* |
| **ClEub R** | AGTTTYATTCTTGCGAACG | *Eubacterium rectale* |
| **g-Blau_F** | GTGAAGGAAGAAGTATCTCGG | *Blautia* genus |
| **g-Blau_R** | TTGGTAAGGTTCTTCGCGTT | *Blautia* genus |
| **314R** | CCTACGGGAGGCAGCAG | *Eubacteria* |
| **518R** | ATTACCGCGGCTGCTGG | *Eubacteria* |

**ST 2 Table. List of Primers used for qPCR analysis**
