# Supplementary material for: Western Indian Rural Gut Microbial Diversity in Extreme Prakriti Endo-Phenotypes Reveals Signature Microbes
Source: Front Microbiol. 2018 Feb 13;9:118. doi: 10.3389/fmicb.2018.00118 (PMC5816807; doi:10.3389/fmicb.2018.00118)
Supplement: Supplementary Table S4 — qPCR analysis data to validate relative abundance of differentially abundant microbial enterotypes in Vata, Pitta, and Kapha. [file Table4.docx]

Table ST4. qPCR analysis data generated to validate relative abundance of differentially abundant microbial enterotypes in Vata, Pitta & Kapha.

**(A): *Eubacteria***

| **Prakriti** | **Ct-value** | **Prakriti** | **Ct-value** | **Prakriti** | **Ct-value** |
| --- | --- | --- | --- | --- | --- |
| Kapha_1 | 10.16 | Pitta_1 | 10.60 | Vata_1 | 10.91 |
| Kapha_2 | 9.34 | Pitta_2 | 10.55 | Vata_2 | 11.84 |
| Kapha_3 | 9.90 | Pitta_3 | 12.96 | Vata_3 | 10.76 |
| Kapha_4 | 9.82 | Pitta_4 | 10.86 | Vata_4 | 11.46 |
| Kapha_5 | 11.18 | Pitta_5 | 12.93 | Vata_5 | 12.83 |
| Kapha_6 | 12.43 | Pitta_6 | 11.75 | Vata_6 | 9.68 |
| Kapha_7 | 11.12 | Pitta_7 | 10.61 | Vata_7 | 11.12 |
| Kapha_8 | 10.06 | Pitta_8 | 13.47 | Vata_8 | 9.58 |
| Kapha_9 | 9.54 | Pitta_9 | 12.83 | Vata_9 | 12.91 |
| Kapha_10 | 12.60 | Pitta_10 | 10.21 | Vata_10 | 10.79 |
| Kapha_11 | 11.27 | Pitta_11 | 10.55 | Vata_11 | 11.12 |
| Kapha_12 | 10.18 | Pitta_12 | 11.17 | Vata_12 | 10.85 |
| Kapha_13 | 10.38 | Pitta_13 | 9.54 | Vata_13 | 11.80 |
| Kapha_14 | 10.47 |  |  | Vata_14 | 10.99 |
| Kapha_15 | 11.10 |  |  | Vata_15 | 9.31 |
| Kapha_16 | 9.90 |  |  | Vata_16 | 11.05 |
| Kapha_17 | 10.16 |  |  | Vata_17 | 9.72 |
| Kapha_18 | 9.34 |  |  |  |  |

**(B) *Blutia sp.***

| **Prakriti** | **Ct-value** | **Prakriti** | **Ct-value** | **Prakriti** | **Ct-value** |
| --- | --- | --- | --- | --- | --- |
| Kapha_1 | No Data | Pitta_1 | 18.07 | Vata_1 | 19.17 |
| Kapha_2 | No Data | Pitta_2 | 20.99 | Vata_2 | 22.25 |
| Kapha_3 | 22.30 | Pitta_3 | 20.75 | Vata_3 | 20.22 |
| Kapha_4 | 21.56 | Pitta_4 | 20.48 | Vata_4 | 18.63 |
| Kapha_5 | 19.18 | Pitta_5 | 19.76 | Vata_5 | 21.85 |
| Kapha_6 | 21.61 | Pitta_6 | 18.70 | Vata_6 | No Data |
| Kapha_7 | 18.83 | Pitta_7 | 18.98 | Vata_7 | 18.99 |
| Kapha_8 | 21.98 | Pitta_8 | 21.53 | Vata_8 | 17.59 |
| Kapha_9 | 19.92 | Pitta_9 | No Data | Vata_9 | 21.04 |
| Kapha_10 | 20.73 | Pitta_10 | No Data | Vata_10 | 19.16 |
| Kapha_11 | 16.97 | Pitta_11 | 19.94 | Vata_11 | 18.73 |
| Kapha_12 | 20.34 | Pitta_12 | 16.73 | Vata_12 | 16.73 |
| Kapha_13 | 20.06 | Pitta_13 | 17.83 | Vata_13 | 19.36 |
| Kapha_14 | 20.09 |  |  | Vata_14 | 19.84 |
| Kapha_15 | 19.65 |  |  | Vata_15 | 17.42 |
| Kapha_16 | No Data |  |  | Vata_16 | 20.84 |
| Kapha_17 | 18.90 |  |  | Vata_17 | No Data |
| Kapha_18 | 19.40 |  |  |  |  |

**(C) *Eubacterium rectale***

| **Prakriti** | **Ct-value** | **Prakriti** | **Ct-value** | **Prakriti** | **Ct-value** |
| --- | --- | --- | --- | --- | --- |
| Kapha_1 | 14.25 | Pitta_1 | 13.00 | Vata_1 | 14.72 |
| Kapha_2 | 13.56 | Pitta_2 | 14.34 | Vata_2 | 15.28 |
| Kapha_3 | 15.44 | Pitta_3 | 15.53 | Vata_3 | 14.24 |
| Kapha_4 | 16.64 | Pitta_4 | 13.92 | Vata_4 | 13.53 |
| Kapha_5 | 13.76 | Pitta_5 | 15.01 | Vata_5 | 16.83 |
| Kapha_6 | 15.31 | Pitta_6 | 15.03 | Vata_6 | 12.78 |
| Kapha_7 | 13.63 | Pitta_7 | 14.65 | Vata_7 | 12.69 |
| Kapha_8 | 16.71 | Pitta_8 | 16.90 | Vata_8 | 11.82 |
| Kapha_9 | 13.78 | Pitta_9 | No Data | Vata_9 | 16.29 |
| Kapha_10 | 15.48 | Pitta_10 | 13.68 | Vata_10 | 14.06 |
| Kapha_11 | 13.58 | Pitta_11 | 16.21 | Vata_11 | 14.52 |
| Kapha_12 | 16.00 | Pitta_12 | 13.92 | Vata_12 | 12.18 |
| Kapha_13 | 13.98 | Pitta_13 | 11.89 | Vata_13 | 15.22 |
| Kapha_14 | 15.57 |  |  | Vata_14 | 13.99 |
| Kapha_15 | 15.35 |  |  | Vata_15 | 10.98 |
| Kapha_16 | No Data |  |  | Vata_16 | 15.26 |
| Kapha_17 | No Data |  |  | Vata_17 | No Data |
| Kapha_18 | No Data |  |  |  |  |

(D) ***Prevotella sp.***

| **Prakriti** | **Ct-value** | **Prakriti** | **Ct-value** | **Prakriti** | **Ct-value** |
| --- | --- | --- | --- | --- | --- |
| Kapha_1 | 13.24 | Pitta_1 | 19.91 | Vata_1 | No Data |
| Kapha_2 | 16.26 | Pitta_2 | 24.94 | Vata_2 | 15.28 |
| Kapha_3 | No Data | Pitta_3 | 25.05 | Vata_3 | 18.49 |
| Kapha_4 | 13.11 | Pitta_4 | 18.59 | Vata_4 | No Data |
| Kapha_5 | 16.05 | Pitta_5 | 27.10 | Vata_5 | No Data |
| Kapha_6 | 16.59 | Pitta_6 | 17.12 | Vata_6 | No Data |
| Kapha_7 | 15.05 | Pitta_7 | 18.80 | Vata_7 | 27.10 |
| Kapha_8 | 12.54 | Pitta_8 | 19.59 | Vata_8 | 13.79 |
| Kapha_9 | 12.92 | Pitta_9 | 20.40 | Vata_9 | 16.95 |
| Kapha_10 | 18.91 | Pitta_10 | No Data | Vata_10 | 15.46 |
| Kapha_11 | No Data | Pitta_11 | 23.37 | Vata_11 | 13.79 |
| Kapha_12 | 12.81 | Pitta_12 | 18.30 | Vata_12 | 14.90 |
| Kapha_13 | 12.95 | Pitta_13 | 17.29 | Vata_13 | 16.34 |
| Kapha_14 | 13.21 |  |  | Vata_14 | 18.44 |
| Kapha_15 | 14.28 |  |  | Vata_15 | 16.75 |
| Kapha_16 | 13.00 |  |  | Vata_16 | 14.51 |
| Kapha_17 | No Data |  |  | Vata_17 | 13.79 |
| Kapha_18 | No Data |  |  |  |  |

**(E) *Roseburia hominis***

| **Prakriti** | **Ct-value** | **Prakriti** | **Ct-value** | **Prakriti** | **Ct-value** |
| --- | --- | --- | --- | --- | --- |
| Kapha_1 | 16.93 | Pitta_1 | 17.94 | Vata_1 | 18.69 |
| Kapha_2 | 16.48 | Pitta_2 | 19.41 | Vata_2 | 18.70 |
| Kapha_3 | No Data | Pitta_3 | 21.61 | Vata_3 | 19.27 |
| Kapha_4 | 19.82 | Pitta_4 | 20.55 | Vata_4 | No Data |
| Kapha_5 | 17.57 | Pitta_5 | 18.76 | Vata_5 | No Data |
| Kapha_6 | 21.91 | Pitta_6 | 19.21 | Vata_6 | No Data |
| Kapha_7 | 16.91 | Pitta_7 | 18.63 | Vata_7 | 16.47 |
| Kapha_8 | 21.44 | Pitta_8 | 21.06 | Vata_8 | 16.56 |
| Kapha_9 | 16.66 | Pitta_9 | No Data | Vata_9 | 18.70 |
| Kapha_10 | 18.89 | Pitta_10 | No Data | Vata_10 | 21.17 |
| Kapha_11 | 14.51 | Pitta_11 | 23.21 | Vata_11 | 16.70 |
| Kapha_12 | 19.15 | Pitta_12 | 22.96 | Vata_12 | 15.54 |
| Kapha_13 | 18.17 | Pitta_13 | 20.78 | Vata_13 | 19.49 |
| Kapha_14 | 19.00 |  |  | Vata_14 | 18.34 |
| Kapha_15 | 17.72 |  |  | Vata_15 | 17.36 |
| Kapha_16 | No Data |  |  | Vata_16 | 17.60 |
| Kapha_17 | No Data |  |  | Vata_17 | 17.27 |
| Kapha_18 | No Data |  |  |  |  |

**Note:** Raw data for qPCR used to estimate Prakriti-specific microbial enterotype.

i) Eubacteria is used as surrogate for total bacterial count (Total sample, n=48). The proportion of specific microbe has been calculated as ratio compared to Eubacteria.

ii) Some parkriti samples had "Undetermined" Ct-value during qPCR for specific bacteria. Those are marked as "No Data".

iii) Each Biological replicate had been used as three Technical replicate during qPCR.

iv) Minimum of n=11 across prakriti-type and microbe-specific has been used during the experiment.
